# How the Organic Food System Supports Sustainable Diets and Translates These into Practice

**DOI:** 10.3389/fnut.2015.00019

**Published:** 2015-06-29

**Authors:** Carola Strassner, Ivana Cavoski, Raffaella Di Cagno, Johannes Kahl, Emmanuelle Kesse-Guyot, Denis Lairon, Nicolas Lampkin, Anne-Kristin Løes, Darja Matt, Urs Niggli, Flavio Paoletti, Sirli Pehme, Ewa Rembiałkowska, Christian Schader, Matthias Stolze

**Affiliations:** ^1^Faculty of Home Economics, Nutritional Science and Facility Management, Münster University of Applied Sciences, Münster, Germany; ^2^Mediterranean Agronomic Institute of Bari (CIHEAM-MAIB), Bari, Italy; ^3^Department of Soil, Plant and Food Science, University of Bari Aldo Moro, Bari, Italy; ^4^Department Organic Food Quality and Food Culture, University of Kassel, Witzenhausen, Germany; ^5^Nutritional Epidemiology Research Team (EREN), Université Paris 13/INRA/INSERM, Bobigny, France; ^6^Nutrition, Obésité, Risque Thrombotique Research Unit (NORT), Aix-Marseille Université, Marseille, France; ^7^The Organic Research Centre, Newbury, UK; ^8^Organic Food and Farming Division, Bioforsk Norwegian Institute for Agricultural and Environmental Research, Tingvoll, Norway; ^9^Estonian University of Life Sciences, Tartu, Estonia; ^10^Department of Socio-Economics, Research Institute of Organic Agriculture (FiBL), Frick, Switzerland; ^11^Council for Agricultural Research and Economics, Research Center on Food and Nutrition, Rome, Italy; ^12^Department of Functional and Organic Food and Commodities, Faculty of Human Nutrition and Consumer Science, Warsaw University of Life Sciences, Warsaw, Poland

**Keywords:** food system, consumption, diet, nutrition, sustainable nutrition, sustainability, health

## Abstract

Organic production and consumption provide a delineated food system that can be explored for its potential contribution to sustainable diets. While organic agriculture improves the sustainability performance on the production side, critical reflections are made on how organic consumption patterns, understood as the practice of people consuming significant amounts of organic produce, may also be taken as an example for sustainable food consumption. The consumption patterns of regular organic consumers seem to be close to the sustainable diet concept of FAO. Certain organic-related measures might therefore be useful in the sustainability assessment of diets, e.g., organic production and organic consumption. Since diets play a central role in shaping food systems and food systems shape diets, the role of organic consumption emerges as an essential topic to be addressed. This role may be based on four important organic achievements: organic agriculture and food production has a definition, well-established principles, public standards, and useful metrics. By 2015, data for organic production and consumption are recorded annually from more than 160 countries, and regulations are in force in more than 80 countries or regions. The organic food system puts the land (agri-cultura) back into the diet; it is the land from which the diet *in toto* is shaped. Therefore, the organic food system provides essential components of a sustainable diet.

## Introduction

The interest in sustainable diets is steadily increasing; for active stakeholders from all agro-food sectors the broader and complex context of the sustainability of food systems is highly relevant for policy and practice (1). Defining a theoretical methodological framework for the assessment of the sustainability of diets presents many challenges. The definition of sustainable diets reached in 2010 at the conference organized by FAO and Biodiversity (2) and the associated four dimensions (health and nutrition, environment, economic, socio-cultural) provide a starting point for a list of indicators serving sustainability assessment (3).

The traditional Mediterranean diet, scientifically well-characterized as a healthy dietary pattern, appreciated for its lower environmental impact and acknowledged as a cultural heritage, is used as a model to assess sustainability of diets and food consumption patterns in the Mediterranean area, using indicators proposed by a Working Group (4).

Members of the International Food Quality and Health Association (FQH) and colleagues explored the organic food system as a case study and its potential support for sustainable diets. FQH experts contributed to a scientific debate on how to address the question of sustainable diets within organic production and consumption concepts and achievements, and what role the organic sector can play to change the global food systems toward a higher degree of sustainability. The aim of the current paper is to resume the output from a seminar arranged in Rome in September 2014 to address these topics, and to explain why the organic food system deserves the role as a good example for developing a global sustainable diet.

## From the Organic Roots Until Today

The context of our diets within food and agriculture systems and the global challenges compel us to address certain issues. Industrial food systems have proven successful in making more food available at a low price. Yet, notwithstanding that some progress has been recently observed, the number of malnourished and hungry people in the world remains unacceptably high (5, 6). At the same time and worldwide, overweight and obesity are increasing among adults and, even more alarmingly, among children (7). The huge amount of food produced is not equitably distributed and, furthermore, roughly one-third of the edible parts of food produced for human consumption are lost or wasted globally (8). In medium- and high-income countries, food is to a great extent wasted, i.e., it is thrown away even if it is still suitable for human consumption. The industrial food systems have developed a strong dependence on fossil energy and caused an undeniable negative effect on the environment (9). Against the background of a looming 9.6 billion people on earth in 2050, many scientists argue for a further intensification of agricultural systems. The organic food system might offer an alternative approach toward sustainable development of the global food system. Even in resource-poor settings, organic management of small farms has been shown to significantly increase yields, improve soil fertility, and water-holding capacity (10–12), enabling a better use of farms’ own resources and reducing dependence on bought inputs (13).

The historical development of the organic food system is relatively young, about 90 years old in Europe, but developing very quickly. The history of the organic movement has a clear and logical sequence: first came the philosophy and teachings, which were based on observation of nature and respect for natural laws. In turn, the organic pioneers transformed these principles into practical farming methods. After development in central Europe, organic agriculture was implemented in nearly all regions throughout the world. Today the organic system is a worldwide food system (14). Such growth is positive; however, it creates a challenge – how to keep high effectiveness together with high quality of produce and basic standards of the organic production having their roots in the pioneer ideas (15).

The organic food system can be described from the organic vision all the way through to the metrics. This includes a food system that raises incomes and increases food security and food safety at both ends; furthermore, as one in which the environment is preserved while farmers and workers have fair access to the means of food production. Today, the system is described in the Codex Alimentarius (16) while the vision is laid down in international standards [e.g., Ref. (17)]. Organic agriculture is defined in a way that reflects the underlying principles (see Box 1). With respect to food, the term *organic* is defined through the principles of organic farming and food production. Organic food quality is defined through process- and product-related aspects (18). There are legal definitions containing regulations in place in Europe (19, 20), Japan (21), and the United States of America (22) both at the national and private standards levels, including a thorough certification process. Indeed, 86 countries around the globe have organic legislation. Evaluation is performed through criteria, indicators, and parameters that can be organic specific. There is a clear connection between the regulation at farm and industry level and the impact on environment and food. In Europe, the organic logo is well recognized by the European consumer and is associated with a sustainable and healthy food system (23–25).

Box 1 **Definition of Organic Agriculture as approved by the IFOAM General Assembly in Vignola, Italy in June 2008.**

“Organic agriculture is a production system that sustains the health of soils, ecosystems, and people. It relies on ecological processes, biodiversity and cycles adapted to local conditions, rather than the use of inputs with adverse effects. Organic agriculture combines tradition, innovation and science to benefit the shared environment and promote fair relationships and a good quality of life for all involved.” [Ref. (17): p. 31].

## On the Dimensions of Sustainability

The framework for Sustainability Assessment of Food and Agriculture (SAFA) systems is used for farms and companies to analyze the sustainability of these organic food chain links (26). Divided into four dimensions (environment, economy, social, and governance), it covers 21 themes and 58 subthemes with defined objectives (27). Preliminary research shows that organic production impacts the entire food system and that organic agriculture can contribute to the main strategies for sustainable food systems: efficiency, consistency, and sufficiency. For a better understanding of this contribution and exploring future scenarios, quantitative models that link food demand, agricultural production patterns, and sustainability impacts are needed (28).

Environmental impacts can be further probed, using the life cycle perspective. As previous studies have shown, in general the agricultural practice has the greatest contribution to environmental impacts of food products (29, 30) but most of the studies focus on the impacts within the farm gate. Hence, environmental effects of transport, processing, and distribution are often excluded. Furthermore, the effect of changing consumption patterns on the effects of the production, such as decreased or increased consumption of meat, is usually not included. The environmental impacts of a diet could be formed as the sum of impacts of each component of the diet, using the life cycle assessment (LCA) approach, a tool to assess the potential environmental impacts, and the use of resources through a product’s life cycle (31). However, there is a lack of studies that include different processing technologies, distribution, and consumption possibilities. These next stages can also have a significant impact, e.g., caused by energy consumption in processing, transport, food losses, and consumer behavior. To enable fair comparison of the environmental impacts of different diets, the selection of functional unit, i.e., the unit in which all impacts are reported, is essential.

Looking to the dimensions of nutrition, economy, society, and culture, key messages include the observation that the consumption of organic food within a diet exhibits certain recurring characteristics, such as a shorter chain in terms of the degrees of separation to the primary producer (32). It is hypothesized that a diet including significant amounts of organic produce is economically fair and affordable, if the diet is sustainable according to the definition (see Box 2 insert below). An alternative indicator to assess the sustainability of diets for the dimension of society and culture might be the degree of training in household skills in schools. This criterion offers potential use for policy formulation. Indeed, education-linked measures, such as food, health, or nutrition literacy, and especially ecoliteracy (33, 34), might offer some further value.

Box 2 **Definition of sustainable diets.**

“Sustainable diets are those diets with low environmental impacts, which contribute to food and nutrition security and to healthy life for present and future generations. Sustainable diets are protective and respectful of biodiversity and ecosystems, culturally acceptable, accessible, economically fair, and affordable; nutritionally adequate, safe, and healthy; while optimizing natural and human resources” (2).

Furthermore, there is growing evidence from profiling organic consumers, e.g., in Germany (35) or France (36), of significantly different dietary choices made by such groups, specifically more healthful choices. Additionally, such data show better nutritional anthropometry measures and physical activity prevalence for organic consumers (37).

A detailed study on the profiles of organic food consumers comprising 54,311 adult participants (36) was presented from the French Nutrinet-Santé cohort (38). The results were in astonishing agreement with those from the German study on organic consumers using data from the National Nutrition Survey II (39, 40). Regular consumers of organic products in both the French and the German cohorts exhibited:

- more plant food based;
- a diet fitting food based and nutritional recommendations;
- markedly less overweight and obesity;
- a higher level of physical activity;
- a non-smoking routine.

Regular consumers of organic products have healthier lifestyle profiles overall and thus a better compliance with the sustainable diet concept [more plant foods, better nutrition, better safety, better lifestyle, and health (adiposity), to minimize energy/water uses and environmental impacts]. Thus, regular consumers of organic products exhibit an overall plant-based diet and a healthier profile better fitting the sustainable diet definition (see Box 2 insert above).

The next steps of the on-going BioNutrinet project will be to precisely quantify organic foods consumption (total and foods/food groups), to evaluate the impacts of five different levels of organic consumption on dietary patterns and nutrient intakes, to determine some impacts of regular organic consumption (versus none), such as energy or water use and environmental pressure using allocated indicators, and to determine pesticide exposure of consumers using pesticide residue analyses in urine/plasma samples. The ultimate goal of this prospective survey will be to assess the impact of organic food consumption on health and well-being parameters.

Applying ecological principles in the pursuit of a sustainable development in agriculture is something that the organic approach shares with the agroecology approach. Using the agroecology perspective of the organic food system shows that, like sustainability, this term also has different meanings to different people, ranging from a purely academic ecology focus to a social movement approach (41–44). The relationship between organic and agro-ecological principles is considered by identifying areas of common ground and of differences (45). The question of how the agro-ecological underpinning of organic farming can be better reflected in organic regulations in future needs to be addressed. The value of certification lies is helping to translate the organic principles into practice through definition of relevant practices and technologies. The concept of certification should be a foundation to support innovation, not a ceiling to constrain it. While it enables markets to reward producers for adopting specific practices, ensuring financial viability of systems, at the same time protecting consumers, it can lead to bureaucracy and institutionalization, disregarding delivery of the broader goals.

## Two Case Studies Provide Insight into Practice in the Organic Food System

The Mediterranean diet is characterized by enormous food diversity where durum wheat is one of the bases, in the form of bread, pasta, couscous, and bulgur. Throughout the Mediterranean different types of bread are made from durum wheat, including traditional flatbreads. A project studying the performance of organically grown old varieties of durum wheat and traditional bread-making provides a case study of linking the organic food system to the Mediterranean diet (46). Organic consumers tend to choose organic breads that are locally produced and handmade, and are processed with natural ingredients. Due to that, traditional sourdough fermentation becomes a very interesting biotechnology for bread making. Even though it produces a smaller loaf size, it is still very appealing to consumers because of its unique characteristics. Recently, there was a valorization of old, local varieties of durum wheat particularly suitable for organic farming. It seems possible to bring traditions and cultural history successfully into modernity. The organic farming practices are undoubtedly a determinant that affects the flour microbiota and, consequently, the dynamic of the sourdough microbial community. The organic cultivation of durum wheat affects flour quality and sourdough fermentation and could be considered a suitable alternative for making sourdough breads with distinctive attributes.

Increasing the consumption of organic food, e.g., by supporting public procurement of organic produce, is a measure that has been extensively utilized, e.g., in Denmark, the country in the world with the largest organic consumption per capita (47). Institutions are often used for a conversion to organic food serving, but schools constitute a sector with a high potential, not least because of the possibility a successful organic food serving implies to influence the habits and preferences of young consumers. The transnational research project iPOPY – innovative Public Organic food Procurement for Youth (2007–2010), one of the eight pilot projects of the CORE-Organic European research area network, was a market research study on how to increase the consumption of organic food in Europe by implementing organic food in school meals. Studies in Denmark, Finland, Italy, Norway, and Germany showed highly variable conditions for school meals, ranging from complete, free meals (Finland) to packed, private lunches (Norway). To maximize organic consumption in schools, complete meals should be served, paid for by the public, with strong public involvement in general, with strong support for organic school food and an adapted supply chain (48). In Finland, the organic proportion was low, but the well-established system has a high potential for significant organic consumption (49). In Italy, significant proportions of organic food were served, supported by public regulations, but hardly communicated (50). Multiple embedding is required to establish a stable, high consumption of organic food in schools. The CORE-Organic I project iPOPY showed that organic food and farming are well suited to discuss and experience sustainability in practice (51). Analysis showed that an organic school policy promotes healthy eating: schools with a healthy food policy also support organic food.

## Conclusion and Outlook

The underlying aim of the organic movement was and is still to create and develop further an alternative food system with focus on primary production (agriculture). To achieve this goal, it is increasingly acknowledged that food processing, distribution, and consumption, including sustainable diets, need to be included in the organic approach. This approach aims at developing a sustainable dietary pattern from field (agriculture) to fork (nutrition). As shown in this paper, what may be called an organic food system offers an instructive example of combining sustainable food production and consumption patterns within one system. However, actually defining an organic food system needs further research and development. Since sustainability issues have been internationally discussed [Rio Earth Summit, Rio 1992; Rio + 5, New York 1997; Rio + 10, Johannesburg 2002; Rio + 20, Rio 2012 etc.], organic agriculture has been placed as an alternative way of production and discussed as a global best practice example. Therefore, during the last decade, many studies and reviews were performed investigating how organic agriculture and food production as well as parts of the value chains contribute to sustainable food production. In parallel, the market for organic food has grown exponentially worldwide. As a consequence, studies on consumer behavior as well as consumption patterns were also carried out. However, until now organic production and consumption have never been brought together in a way describing the complete organic food system. Furthermore, the link to sustainable diets is implicated but, as the seminar in Rome concluded, needs further investigation. While organic agriculture can be taken as an example for sustainable food production, critical reflections were made on how organic consumption patterns may also be taken as example for sustainable food consumption. One of the inputs organic may offer here are parameters where organic-related measures might be useful in the sustainability assessment of diets and the notion of characterizing organic value chains from a sustainability perspective on the basis of the dimensions discussed (health and nutrition, environment, economic, socio-cultural). Two potential indicators considered more closely were for organic production (land use under organic cultivation in percentage) and for organic consumption (organic consumption per capita). Drawing on the definition of sustainable diets above, the change of consumption patterns seems to be a crucial issue in the transformation to sustainable food systems. The consumption patterns of regular organic consumers seem to be close to the sustainable diet concept of FAO. Since diets play a central role in shaping food systems and food systems shape diets, the role of organic food consumption in sustainable diets emerges as an essential topic to be addressed. An argument for using the organic sector as an example to learn from while designing a sustainable diet is the viability of delineating the organic food system as a living laboratory. This laboratory has a clear and generally accepted definition, four basic principles, internationally acknowledged standards and it has useful and well-established metrics. The organic food system has been in practice for the last 100 years and covers environmental aspects, animal welfare standards, and food quality as well as health issues. So, today it also has the data from more than 160 countries, and regulations are in force in more than 80 countries or regions. The organic food system puts the land (agri-cultura) back into the diet; indeed, it is the land from which the diet *in toto* is shaped. Therefore, the organic food system provides the essential requirements of a sustainable diet. An international multidisciplinary project is being prepared to develop the concept of an organic diet more in depth and evaluate how it plays a key role in the organic food system as an important component of sustainable food systems.

## Conflict of Interest Statement

The authors declare that the research was conducted in the absence of any commercial or financial relationships that could be construed as a potential conflict of interest.
